# Correlation between whole salivary prostaglandin E_2_ and hemoglobin A1c levels among type-2 diabetic and non-diabetic patients with periodontal inflammation

**DOI:** 10.1186/s12903-024-04032-z

**Published:** 2024-02-23

**Authors:** Marwa Y. Shaheen, Amani M. Basudan, Abeer S. Alzawawi, Fatemah Al-Ahmari, Hajer A. Aldulaijan, Nouf Alshibani, Rakan Saifuddin Shaheen, Reem Al-kattan

**Affiliations:** 1 Department of Periodontics and Community Dentistry, College of Dentistry, King Saud University, Riyadh, 11545 Saudi Arabia; 2 Periodontics Division, Department of Preventive Dentistry, College of Medicine and Dentistry, Riyadh Elm University, Riyadh, Saudi Arabia

**Keywords:** Alveolar bone loss, Hemoglobin A1c, Periodontal inflammation, Prostaglandin E2, Type-2 diabetes mellitus, Unstimulated whole saliva

## Abstract

**Background:**

It is hypothesized that whole salivary prostaglandin E_2_ (PgE_2_) levels are higher in patients with type-2 diabetes mellitus (type-2 DM) than non-diabetic individuals with periodontal inflammation; and that whole salivary expression of PgE_2_ is correlated with hemoglobin A1C (HbA1c) levels. The aim of the present study was to compare whole salivary PgE_2_ levels among patients with type-2 DM and non-diabetic individuals with periodontal inflammation.

**Methods:**

Sociodemographic data, duration since the diagnosis and management of type-2 DM, most recent hemoglobin A1C (HbA1c level), and any familial history of DM was retrieved from patient’s healthcare records. Participants were divided into four groups: Group-1: type-2 diabetics with periodontal inflammation; Group-2: type-2 diabetics without periodontal inflammation; Group-3: non-diabetics with periodontal inflammation; and Group-4: non-diabetics without periodontal inflammation. Plaque and gingival indices (PI and GI), probing depth (PD), clinical attachment loss (CAL) and marginal bone loss (MBL) were measured. Unstimulated whole saliva samples were collected and PgE_2_ levels were measured. Group-comparisons were done and *P* < 0.05 were considered statistically significant.

**Results:**

One-hundred-sixty individuals were included. Mean HbA1c levels were higher in Group-1 than groups 2 (*P* < 0.05), 3 (*P* < 0.05) and 4 (*P* < 0.05). The PI (*P* < 0.05), GI (*P* < 0.05) and PD (*P* < 0.05) were higher in Group-1 than groups 2 and 4. The CAL was higher in Group-1 than groups 2 (*P* < 0.05) and 3 (*P* < 0.05). The PD (*P* < 0.05), PI (*P* < 0.05) and GI (*P* < 0.05) were higher in Group-3 than Group-4. The MBL was higher in Group-1 than groups 2 (*P* < 0.05), 3 (*P* < 0.05) and 4 (*P* < 0.05). The PgE_2_ levels were higher in Group-1 than groups 2 (*P* < 0.05), 3 (*P* < 0.05) and 4 (*P* < 0.05).

**Conclusion:**

Hyperglycemia in patients with type-2 DM is associated with increased expression of whole salivary PgE2 levels and worsened periodontal inflammation compared with individuals with well-controlled type-2 DM and non-diabetic individuals.

## Introduction

Periodontal inflammation, a common oral health condition presents with clinical signs and symptoms including gingival erythema, gingival bleeding and probing depth (PD) ≥ 3 mm [1]. If left undiagnosed and untreated in a timely fashion, unaddressed inflammation of the periodontal soft tissues may advance, resulting in the deterioration of bone structures within the dental socket, eventually resulting in tooth loss [2]. A compromised oral hygiene status is a classical yet local risk factor of periodontal inflammation [3]; however, from a systemic perspective persistent hyperglycemia (PH) is a noteworthy risk factor of periodontal inflammation. A state of PH is a hallmark of poorly-controlled type-2 diabetes mellitus (type-2 DM) [4–7]. Hemoglobin A1c (HbA1c), a glycated hemoglobin molecule formed by the non-enzymatic attachment of glucose to hemoglobin in erythrocytes, serves as a valuable biomarker for assessing long-term glycemic control in individuals with DM [8]. The HbA1c measurement reflects the average blood glucose levels over the preceding two to three months [8]. Studies have shown that scores of periodontal inflammatory parameters including plaque index (PI), gingival index (GI), PD and clinical attachment loss (CAL) are elevated in patients with type-2 diabetic patients with poor metabolic control in contrast to patients with well-controlled type-2 DM and non-diabetic individuals [6]. Moreover, from an immunoinflammatory perspective, PH increases the production of advanced glycation endproducts (AGEs) and destructive inflammatory cytokines such as interleukin (IL) 1 beta, IL-6, and tumor necrosis factor alpha in oral biologic fluids such as unstimulated whole saliva (UWS) [9–12].

Prostaglandin E_2_ (PgE_2_) is a bioactive lipid molecule that plays a pivotal role in a wide range of physiological and pathological processes within the human body [13–15]. Its diverse functions include regulation of inflammation, immune response, vasodilation, and pain perception [13, 16, 17]. The biologic activity of PgE_2_ chiefly relies on four G-protein-coupled receptors (EP1-4), each activating distinct intracellular signaling pathways [18]. According to Li et al. [18], PgE_2_ plays a critical role in regulating renal water transport through various mechanisms. Likewise, in a experimental study on mice, Yasui et al. [19] reported that PgE2 exerts a pivotal function in inflammation of adipose tissue associated with obesity and insulin resistance by overseeing the recruitment and polarization of macrophages. Due to its multifaceted influence on numerous physiological pathways, PgE_2_ holds a central place in both basic research and clinical applications, making it a fascinating molecule to explore in the realm of biochemistry and medicine. In periodontal research, a limited number of studies have correlated periodontal inflammation with expression of PgE_2_ in UWS [20–22]. For instance in the study by Sánchez et al. [22] whole salivary PgE_2_ levels were significantly higher in patients with than without periodontitis. In this study [22], a statistically significant effect size was also reported between PgE_2_ and PD (*P* < 0.01). Similarly, another study [20] evaluated whole salivary PgE_2_ levels among patients with generalized chronic periodontitis. The results showed that whole salivary PgE_2_ levels are significantly higher in patients with than without periodontitis with a significantly correlation with PI [20]. However, a vigilant review of pertinent indexed literature has shown that to date, there are no studies that have assessed PgE_2_ levels in UWS samples obtained from patients with type-2 DM.

The present study is based on the hypothesis that whole salivary PgE_2_ levels are higher in type-2 diabetic than non-diabetic patients with periodontal inflammation; and that whole salivary expression of PgE_2_ is correlated with HbA1c levels. The aim of the present study was to compare whole salivary PgE_2_ levels among type-2 diabetic and non-diabetic patients with periodontal inflammation.

## Materials and methods

### Ethical approval

The study protocol was reviewed and approved by the College of Medicine Research Ethics Committee at King Saud University, Riyadh, Saudi Arabia (Ref # 23/0663/IRB; Approval # E-23-8102). The study was registered and approved by the College of Dentistry Research Center (CDRC) at King Saud University, Riyadh, Saudi Arabia (CDRC No. FR0695). Participation in the study was entirely optional, and all individuals had the option to discontinue their involvement at any stage during the research. Participants demographics such as name, address, and contact details will be kept confidential. All volunteering participants were encouraged to pose any questions or inquiries they had. Those who chose to volunteer were asked to carefully review and sign a written informed consent document.

### Study location and design

The study was performed at the Department of Periodontics and Community Dentistry, King Saud University, Riyadh, Saudi Arabia. The present investigation had a cross-sectional comparative study in which, patients with and without medically diagnosed type-2 DM were included.

### Inclusion and exclusion criteria

The inclusion criteria were as follows: (a) adult individuals (aged ≥ 18 years); (b) patients with medically diagnosed type-2 DM as per medical records; (c) self-reported non-diabetic individuals; (d) signing the consent form; (e) diagnosis periodontal inflammation [1]; (e) patients with a healthy periodontal status [1]; and (f) HbA1c levels measurement within the past 60 days. The following exclusion criteria were imposed: (a) self-reported tobacco-smokers and smokeless tobacco product users; (c) individuals with self-reported systemic diseases other than type-2 diabetes mellitus such as prediabetes, cardiovascular disorders, renal and hepatic disorders, and HIV/AIDS individuals; (d) pregnant and/or nursing females and habitual alcohol users. Moreover, from a dental perspective, edentulous individuals, third molars, supernumerary teeth and grossly carious teeth with embedded root remnants will not be evaluated and will be considered as “missing teeth”.

### Evaluation of patients’ medical records

An evaluation was conducted on patients’ digital healthcare records/charts by the principal investigator to extract pertinent information pertaining to the patients’ age, gender, duration since the diagnosis and management protocol of type-2 DM, most recent HbA1c level, and any familial history of DM.

### Study groups

Participants were divided into four groups: Group-1: type-2 diabetic patients with periodontal inflammation; Group-2: type-2 diabetic patients without periodontal inflammation; Group-3: non-diabetic patients with periodontal inflammation; and Group-4: non-diabetic patients without periodontal inflammation.

### Clinical and radiographic periodontal examination

In all patients, the following clinical periodontal parameters were assessed: (a) PI [23]; (b) GI [24]; (c) PD [25] and (d) CAL [26]. The PI and GI were measured on four sites per tooth (mesial, distal, lingual/palatal and facial/buccal). The PD was assessed on six sites per tooth (mesiobuccal, midbuccal, distobuccal, mesio lingual/palatal, midlingual/palatal and distolingual/palatal). The PD and CAL were measured using a sterile graded probe (UNC 15, Chicago, IL, USA). Marginal bone loss (MBL) was measured on mesial and distal surfaces of all teeth using digital intraoral radiographs that were taken using the ling cone paralleling technique. The MBL was recorded as the linear distance from two millimeters (mm) below the cementoenamel junction to the alveolar crest [6]. All clinical and radiographic evaluations were done by one blinded, trained and calibrated investigator (*Kappa* scores 0.88 and 0.9, respectively).

### Collection of unstimulated whole saliva samples and assessment of prostaglandin E_2_ levels

The UWS samples were collected from type-2 diabetic and non-diabetic patients as described elsewhere [10, 27]. In summary, participants were informed about the procedure of UWS collection and were instructed to refrain from eating, and/or drinking for at least 2 h before UWS collection. The participants were seated in a quiet room and will be requested to allow saliva to accumulate in the mouth for five continuous minutes. Participants were instructed to refrain from jaw movements and swallowing during this time period. At the end of 5-minutes, participants were requested to expectorate the UWS into a measuring cylinder through a disposable plastic funnel. The unstimulated whole salivary flow rate was immediately calculated. The UWS samples were immediately transferred to sterile plastic tubes with lid and centrifuged at 2000xg for 15 min in a cold room (4^o^C). The supernatant was immediately collected and stored at -70 ^o^C. All UWS supernatants were assessed for PgE_2_ levels within 24 h of collection. Collection of UWS and related supernatants samples were carried out by one blinded investigator (Kappa scores 0.88 and 0.86, respectively). Whole salivary PgE_2_ levels were measured as described in a previous study [21]. In summary, whole salivary PgE_2_ levels were analyzed in duplicate wells using a human PgE_2_ sandwich enzyme linked immunosorbent assay kit (Biosource, Invitrogen Corporation, Carlsbad, CA, USA). The kit was in accordance with the manufacturers’ instructions. The laboratory-based investigations were done by a calibrated investigator blinded to the study groups (*Kappa* score 0.92).

### Sample-size estimation and statistical analyses

Sample-size estimation was performed on data obtained from a pilot investigation (*n*Query Advisor 6.0, Statistical Solutions, Saugas, MA., USA). The desired level of statistical power was set at 0.80, indicating an 80% chance of detecting a true effect if it exists, and an alpha of 5%, representing the threshold for statistical significance. In order to detect a difference of 2 mm in PD in the study groups, it was estimated that at least 32 patients should be included in each group. All statistical analyses were done using a computer software (SPSS, version 22, Chicago, IL, USA). The Shapiro-Wilk test was used to assess data normality. The means and standard deviations for all clinical, radiographic and laboratory-based parameters were determined; and group comparisons will be done using the One-way Analysis of Variance and Bonferroni *Post-hoc* adjustment tests. Correlations between clinico-radiographic parameters and whole salivary PgE_2_ levels were assessed using logistic regression models. *P*-values less than 0.05 were considered statistically significant. Intra-examiner reliability will be assessed through repeated measurements on a subset of patients by calculation of kappa score.

## Results

### Study cohort

**Fig. 1:**
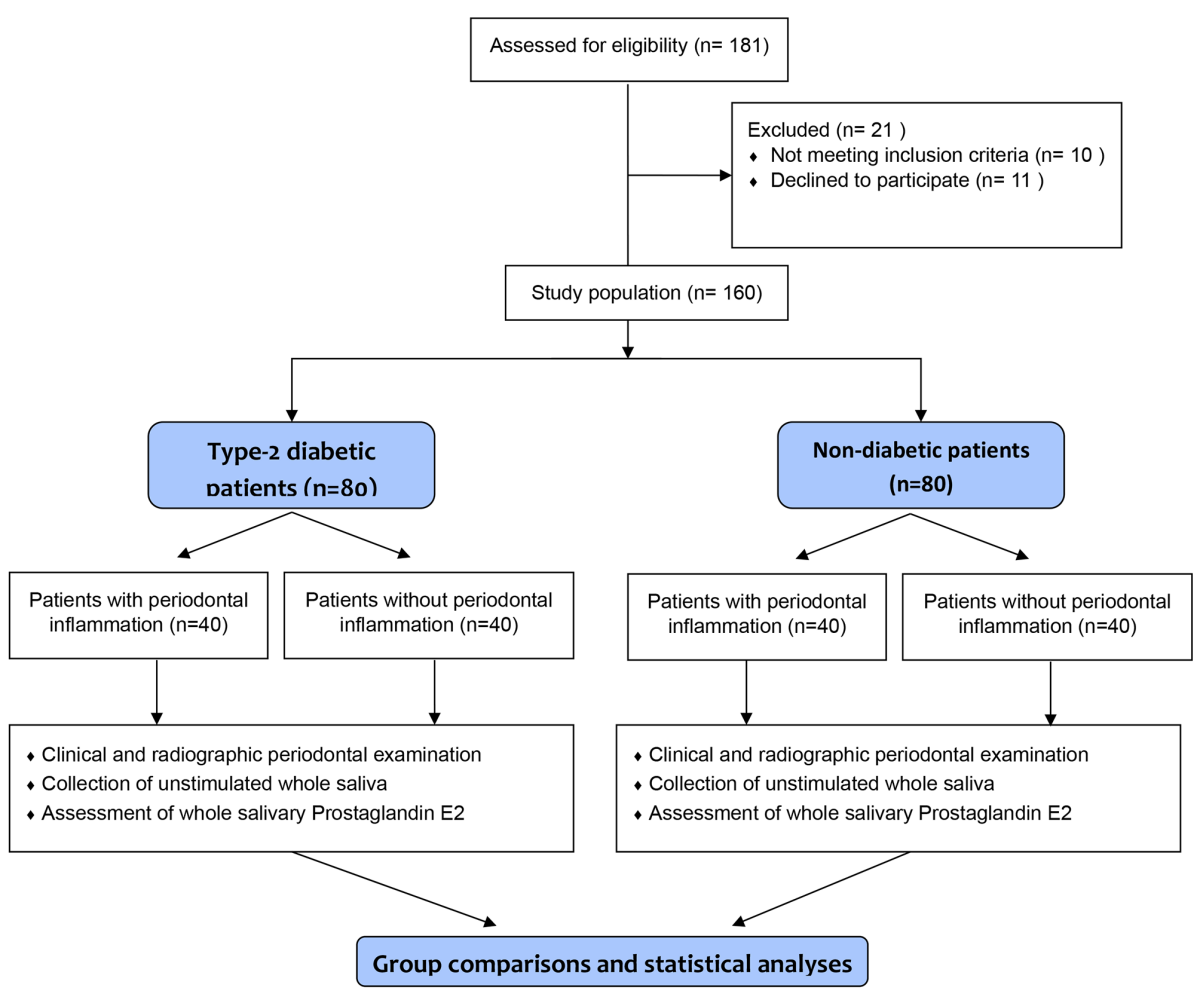
Flow diagram

An initial cohort comprising 181 individuals was extended invitations to partake. Subsequently, 21 individuals who failed to meet the predetermined eligibility criteria were subsequently excluded from the study. Consequently, the study encompassed a final sample size of 160 individuals, stratified into two groups: 80 individuals with a diagnosis of type-2 DM, and an equivalent number of 80 individuals without type-2 DM. Within the subset of type-2 DM-afflicted individuals, 40 individuals exhibited clinical manifestations of periodontal diseases, while the remaining 40 exhibited a state of periodontal health. In the non-diabetic control group, 40 individuals exhibited a state of periodontal health, while the remaining 40 presented clinical evidence of periodontal inflammation, as illustrated in Fig. 1. All type-2 diabetic patients had been prescribed orally ingested anti-hyperglycemic medications and were also instructed to observe dietary control.

In groups 1, 2, 3 and 4, 25, 30, 33 and 30 participants were male, respectively. There was no significant difference in the mean age of individuals in all groups. The mean HbA1c levels were significantly higher in Group-1 (7.6 ± 0.4) compared with groups 2 (5.1 ± 0.5) (*P* < 0.05), 3 (4.9 ± 0.2) (*P* < 0.05) and 4 (4.6 ± 0.2) (*P* < 0.05). The duration of type-2 DM in groups 1 and 2 was 5.7 ± 1.4 and 5.02 ± 0.9 years, respectively. There was no difference in the mean HbA1c levels among patients in groups 2, 3 and 4. A family history of DM was more often reported by patients in Group-1 (40%) compared with patients in groups 2 (12.5%) and 3 (7.5%). None of the participants in Group-4 had a family history of DM. Toothbrushing twice daily was more often reported by participants in Group-4 compared with individuals in groups 1 (20%), 2 (50%) and 3 (25%). Once daily flossing of interproximal spaces was reported by 10% and 52.5% individuals in groups 2 and 4, respectively. None of the participants in groups 1 and 3 reported to have ever used dental floss (Table 1).

**Table 1:** Demographics of the study population

Parameters	Group-1	Group-2	Group-3	Group-4
Participants (n)	40	40	40	40
Gender (male)	25	30	33	30
Mean age in years	53.3 ± 5.5 years	50.1 ± 7.9 years	51.1 ± 7.9 years	48.4 ± 12 years
Hemoglobin A1c	7.6 ± 0.4^*†‡^	5.1 ± 0.5	4.9 ± 0.2	4.6 ± 0.2
Duration of type-2 DM	5.7 ± 1.4 years^*^	5.02 ± 0.9 years	NA	NA
Family history of DM (%)	16 (40%)	5 (12.5%)	3 (7.5%)	None
Twice daily toothbrushing (%)	8 (20%)	20 (50%)	10 (25%)	36 (90%)
Once daily flossing (%)	—	4 (10%)	—	21 (52.5%)

DM: Diabetes mellitus ^*^Compared with Group-2 (*P* < 0.05); ^†^Compared with Group-3 (*P* < 0.05); ^‡^Compared with Group-4 (*P* < 0.05)

### Clinical and radiographic parameters

Scores of PI (*P* < 0.05), GI (*P* < 0.05) and PD (*P* < 0.05) were significantly higher among patients in Group-1 compared with individuals in groups 2 and 4. Score of CAL was significantly higher among patients in Group-1 compared with individuals in groups 2 (*P* < 0.05) and 3 (*P* < 0.05). Scores of PI (*P* < 0.05) and GI (*P* < 0.05) were significantly higher among patients in Group-3 compared with individuals in Group-2. The PD (*P* < 0.05), PI (*P* < 0.05) and GI (*P* < 0.05) were significantly higher in Group-3 compared with Group-4. The mesial and distal MBL was significantly higher in Group-1 compared with groups 2 (*P* < 0.05), 3(*P* < 0.05) and 4 (*P* < 0.05). There was no significant difference in MBL among patients in groups 2, 3 and 4. There was no statistically significant difference in clinical and radiographic parameters among patients in groups 2 and 4 (Table 2).

**Table 2:** Periodontal parameters

Parameters	Group-1	Group-2	Group-3	Group-4
Plaque index	0.78 ± 0.18^*†^	0.11 ± 0.12^§^	0.65 ± 0.2^‖^	0.09 ± 0.03
Gingival index	0.7 ± 0.31^*†^	0.08 ± 0.14^§^	0.66 ± 0.27^‖^	0.07 ± 0.02
Probing depth	4.73 ± 0.86 mm^*†^	1.54 ± 0.77 mm^§^	3.73 ± 0.67 mm^‖^	0.8 ± 0.6 mm
Clinical attachment loss	3.21 ± 0.8 mm^*‡^	0.61 ± 0.41 mm	0.6 ± 0.44 mm	—
Marginal bone loss (mesial)	4.31 ± 0.71 mm^*†‡^	1.9 ± 0.56 mm	1.43 ± 0.78 mm	0.61 ± 0.5 mm
Marginal bone loss (distal)	4.27 ± 0.47 mm^*†‡^	1.74 ± 0.61 mm	1.31 ± 0.69 mm	0.52 ± 0.4 mm

mm: millimeters ^*^Compared with Group-2 (*P* < 0.05) ^†^Compared with Group-4 (*P* < 0.05) ^‡^Compared with Group-3 (*P* < 0.05) ^§^Compared with Group-3 (*P* < 0.05) ^‖^Compared with Group-4 (*P* < 0.05)

### Unstimulated whole salivary flow rate and PgE2 levels

The UWSFR was significantly higher among patients in groups 2 (*P* < 0.05), 3 (*P* < 0.05) and 4 (*P* < 0.05) compared with patients in Group-1. There was no statistically significant difference in UWSFR among patients in groups 2, 3 and 4. The whole salivary PgE_2_ levels were significantly higher in Group-1 compared with groups 2 (*P* < 0.05), 3 (*P* < 0.05) and 4 (*P* < 0.05). The whole salivary PgE_2_ levels were significantly higher in Group-3 compared with groups 2 (*P* < 0.05), and 4 (*P* < 0.05). There was no difference in whole salivary PgE_2_ levels among patients in groups 2 and 4 (Table 3).

**Table 3:** Periodontal parameters

Parameters	Group-1	Group-2	Group-3	Group-4
Unstimulated whole salivary flow rate	0.08 ± 0.03 ml/min^*^	0.37 ± 0.05 ml/min	0.33 ± 0.02 ml/min	0.32 ± 0.01 ml/min
Prostaglandin E2 level	202.6 ± 66.71 pg/ml^†^	31.5 ± 9.77 pg/ml	96.9 ± 39.5 pg/ml^‡^	24.02 ± 10.6 pg/ml

pg/ml: picograms per milliliter ^*^Compared with groups 2 (*P* < 0.05), 3 (*P* < 0.05) and 4 (*P* < 0.05). ^†^Compared with groups 2 (*P* < 0.05), 3 (*P* < 0.05) and 4 (*P* < 0.05). ^‡^ Compared with groups 2 (*P* < 0.05) and 4 (*P* < 0.05)

### Regression analysis

**Fig. 2:**
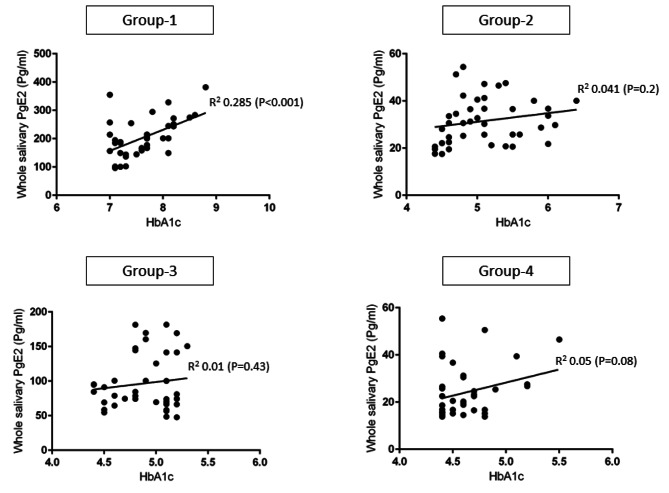
Correlation between hemoglobin A1c levels and whole salivary PgE_2_ levels

**Fig. 3:**
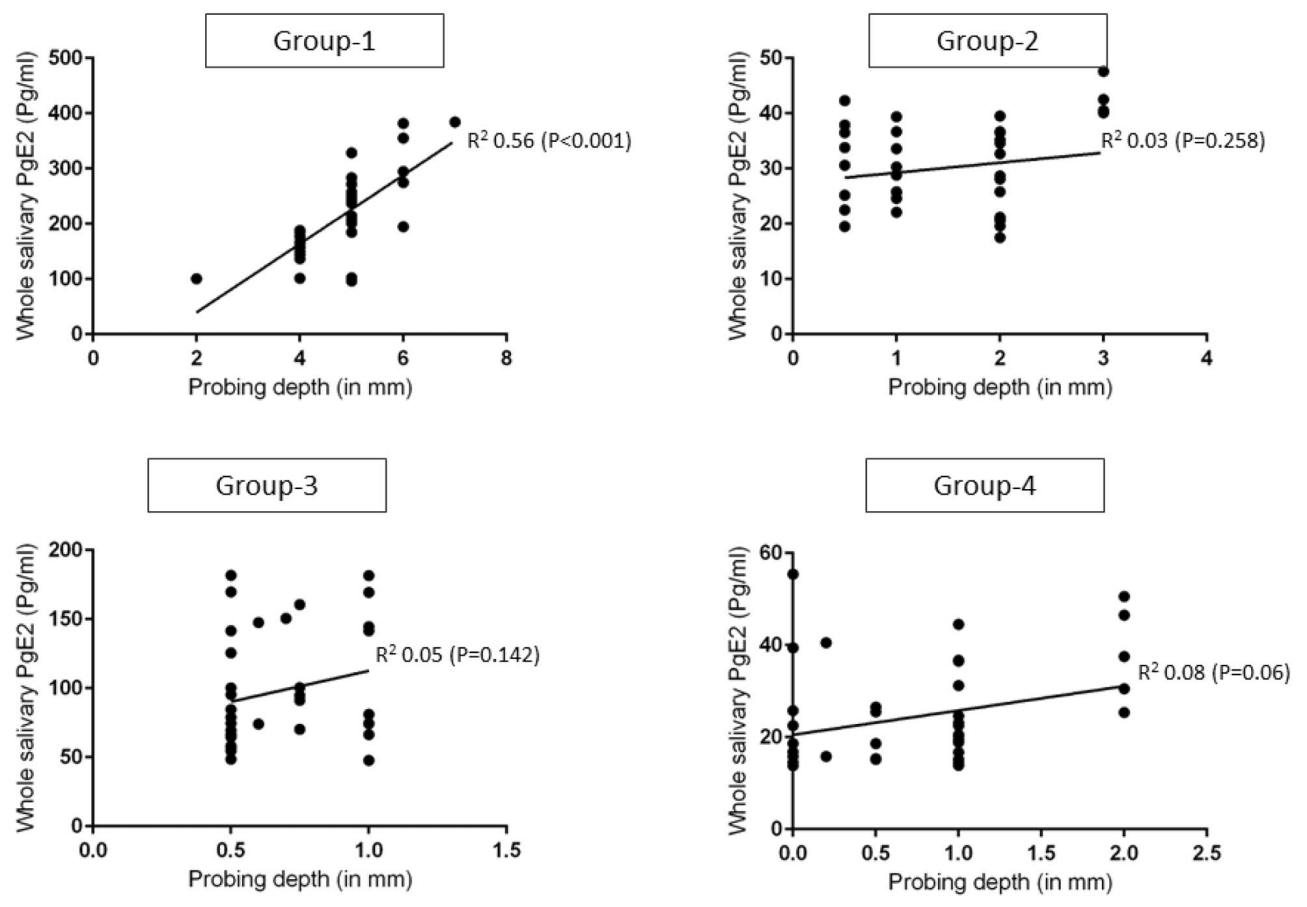
Correlation between probing depth (in millimeters) and whole salivary PgE_2_ levels

In Group-1. there was a statistically significant correlation between HbA1c levels (*P* < 0.001) and PD (*P* < 0.001) and whole salivary PgE_2_ levels in comparison with groups 2, 3 and 4 (Figs. 2 and 3). There was no statistically significant correlation between age, gender, duration of type-2 DM, family history of DM, UWSFR, PI, GI, CAL, MBL and oral hygiene maintenance (OHM) protocols and whole salivary PgE_2_ levels.

## Discussion

Collectively, the clinical and radiographic results of the present observational study are consistent with previous investigations [28–31] as clinical and radiographic markers of periodontal inflammation were worse in patients with poorly-controlled type-2 DM (Group-1) compared with systemically healthy individuals (Group-4). It is noteworthy that HbA1c levels of patients in Group-1 were significantly higher than those among groups 2, 3 and 4. These observations serve as a reaffirmation of the established scientific knowledge that a persistent state of hyperglycemia, which frequently manifests in patients with poorly controlled DM, exacerbates inflammation through the upregulation of AGEs within various biological fluids, including serum, saliva, and gingival crevicular fluid (GCF) [12, 32, 33]. In a recent experimental study on mouse models Tang et al. [34] assessed the effect of persistent hyperglycemia on periodontal tissues. The results showed that a state of persistent hyperglycemia induces oxidative stress (OS) in periodontal tissues including the periodontal ligament, which augments alveolar bone loss [34]. Here, it is also pertinent to refer to a recent experimental study in which Liu et al. [35] investigated the role of the PgE_2_ pathway in mediating tissue damage. Results by Liu et al. [35] showed that PgE_2_ pathways mediate OS in systemic epithelial cells. Additionally, in a molecular cross-sectional epidemiological study conducted by Zhong et al. [36], the authors examined the associations between the mean concentrations of PgE_2_ in the GCF and clinical signs of periodontal disease within a community-based sample encompassing 6277 adults with ages ranging from 52 to 74 years. The results showed that the GCF PgE_2_ levels were positively correlated with periodontal PD and gingival bleeding in the study population [36]. Authors of the present investigation applaud the results reported by Zhong et al. [36] as the periodontal inflammatory parameters were worse and whole salivary PgE_2_ levels were significantly higher in group-1 compared with other groups. Moreover, in support of the results reported by Zhong et al. [36], the present results also demonstrated a statistically significant correlation between whole salivary PgE_2_ levels and PD in Group-1 compared with other groups (Fig. 3). A remarkable observation in the present study was that a statistically significant correlation as noted between whole salivary PgE2 levels and HbA1c levels in Group-1 as shown in Fig. 2. This suggests that rising HbA1c levels modulate the expression of PgE_2_ in bodily fluids including UWS. It is therefore speculated that a state of persistent hyperglycemia among patients in Group-1 induced a state of OS in the periodontal tissues of these individuals thereby exacerbating the clinical and radiographic markers of periodontal inflammation and enhancing the expression of whole salivary PgE_2_. Interestingly, the present results showed no statistically significant difference in clinical and radiographic periodontal parameters among patients in groups 2 (type-2 diabetic patients without periodontal inflammation) and 4. In this context, it is hypothesized that assessment of PgE_2_ in UWS is a potential marker of periodontal disease in predominantly undiagnosed cases. Further studies are warranted to test this hypothesis.

It is noteworthy that the type-2 diabetic patient population in Group-2 demonstrated periodontal statuses, unstimulated whole salivary flow rates and whole salivary PgE2 levels similar to non-diabetic patients with a healthy periodontal status (Group-4). One possible rationale in this regard is that individuals in the former group exhibited an average HbA1c level of 5%, indicating that these individuals had well-controlled type-2 DM, resulting in HbA1c levels that were statistically similar to those of non-diabetic patients (Group-4). The current findings further corroborate earlier investigations [37, 38] that underscore the critical role of glycemic control in preserving a consistent periodontal health status among individuals with clinically diagnosed DM. It is highly recommended that community-based health education programs should routinely be carried out to educate the masses about the association between systemic diseases such as DM and oral inflammatory diseases including periodontal diseases. Moreover, such educational programs should also emphasize the importance of understanding the adverse consequences of elevated blood sugar levels (hyperglycemia) and the advantages of maintaining glycemic control in relation to periodontal health and overall well-being.

One limitation of the present investigation is that subgingival microbiota remained uninvestigated in the study groups. It is speculated that subgingival microbial counts such as red complex bacteria are higher in patients with poorly-controlled type-2 DM in comparison with individuals with well-controlled type-2 DM and non-diabetic individuals. Since poorly-controlled DM is associated with delayed healing, it is speculated that the outcomes of periodontal interventions such as surgical and non-surgical periodontal therapy (NSSPT) are compromised in patients with poorly-controlled type-2 DM than among individuals with individuals with well-controlled type-2 DM and non-diabetic individuals. Furthermore, individuals using combustible and non-combustible nicotine products were excluded in the present investigation. It is well-established that habitual use of nicotinic products is associated with an increased risk of periodontal diseases compared with individuals not using any tobacco product [39, 40]. Such habits have also been reported to jeopardize the efficacy of periodontal interventions such as NSSPT [40]. It is therefore likely that whole salivary PgE_2_ levels are altered in smokers as compared to non-smokers thereby augmenting inflammation in the former group. Further well-designed studies are needed to test these hypotheses. It is notable that toothbrushing twice daily and flossing of interdental spaces was performed more often by patients in groups-2 and 4 compared with individuals in Group-1. It has been reported that a compromised education status and unprivileged living standards are risk-factors of poorly-controlled DM as well as periodontal diseases. It is hypothesized that improvements in lifestyle and education among diabetic patients helps improve their oral and systemic health statuses. Further studies are needed to test these hypotheses.

## Conclusion

Hyperglycemia in patients with type-2 DM is associated with increased expression of whole salivary PgE2 levels and worsened periodontal inflammation compared with individuals with well-controlled type-2 DM and non-diabetic individuals.

## Data Availability

The datasets used and/or analysed during the current study are available from the corresponding author on reasonable request.
